# Nevertheless, partisanship persisted: fake news warnings help briefly, but bias returns with time

**DOI:** 10.1186/s41235-021-00315-z

**Published:** 2021-07-23

**Authors:** Rebecca Hofstein Grady, Peter H. Ditto, Elizabeth F. Loftus

**Affiliations:** Department of Psychological Science, University of California, Irvine, 4201 Social and Behavioral Sciences Gateway, Irvine, CA 92697-7085 USA

**Keywords:** Partisanship, Politics, Fake news, Misinformation, False memory

## Abstract

Politically oriented “fake news”—false stories or headlines created to support or attack a political position or person—is increasingly being shared and believed on social media. Many online platforms have taken steps to address this by adding a warning label to articles identified as false, but past research has shown mixed evidence for the effectiveness of such labels, and many prior studies have looked only at either short-term impacts or non-political information. This study tested three versions of fake news labels with 541 online participants in a two-wave study. A warning that came before a false headline was initially very effective in both discouraging belief in false headlines generally and eliminating a partisan congruency effect (the tendency to believe politically congenial information more readily than politically uncongenial information). In the follow-up survey two weeks later, however, we found both high levels of belief in the articles and the re-emergence of a partisan congruency effect in all warning conditions, even though participants had known just two weeks ago the items were false. The new pre-warning before the headline showed some small improvements over other types, but did not stop people from believing the article once seen again without a warning. This finding suggests that warnings do have an important immediate impact and may work well in the short term, though the durability of that protection is limited.

**Supplementary Information:**

The online version contains supplementary material available at 10.1186/s41235-021-00315-z.

## Statement of significance

This research is intended to test the effectiveness of real-world warning labels being used by major social media companies to help reduce the problem of politically driven “fake news” on their platforms. These platforms have tried various methods to get people to recognize and reject false stories, but they have not always been effective. This study looks at one potential improvement, moving warning labels to *before* the headline is presented, instead of with it, or presenting a correction after it. Past research in other areas has shown that this can increase the effectiveness of such labels, and Facebook recently added something like this to some fact-checked articles. However, there is a strong tendency for people to hold on to beliefs, even if false, that support their political leanings, and that information that is discredited can continue to affect people over time. We found that presenting the warning before a false headline was effective initially, though it was not significantly better in most areas than the label under the headline. But two weeks later, across conditions, people once again believed items they once knew were false, especially when those items supported their political views. The new pre-warning before the headline showed some small improvements over other types, but did not stop people from believing the article once seen again without a warning. This shows that warnings may have a short-term impact, which may help reduce how much the misinformation is spread, though they are not effective of an inoculation over time.

## Introduction

There is a dangerous and growing distribution of misinformation online around important topics such as vaccine effectiveness, electoral fraud, and political conspiracies (Kavanagh & Rich, 2018). This is especially true on social media, where the low barrier to entry and algorithm-driven distribution that prioritizes views and clicks over accuracy drives the high spread of fake news on such platforms (Martens et al., 2018). Recent concerns have revolved around the impact of doctored photographs and videos. For example, a video of Speaker of the House Nancy Pelosi slowed down to make her look drunk was removed by some websites, while others left it on their platform (Roettgers, 2019). Unfortunately, fact-checking and corrections are not always enough to counteract people’s belief in false information. Past studies have suggested that corrections may even backfire and cause increased belief in that information (Nyhan & Reifler, 2010), though recent research has found evidence against such an impact (e.g., see Ecker et al., 2020; Swire-Thompson et al., 2020). This persistence of the original misinformation, even after a warning or a correction, is often referred to as the Continued Influence Effect or Belief Perseverance, in that previously believed information that is learned to be suspect continues to affect later judgments (Anderson et al., 1980; Lewandowsky et al., 2012).

In recent years, social media companies like Facebook have been partnering with fact-checking organizations to combat the spread of misinformation. Many sites do not want to block the sharing of articles, even if they have been identified as false by fact-checkers, because they do not want to infringe on the free speech rights of their users or become the “arbiter of truth” in making the determination of what is true or false (Levi, 2017). Instead of outright removal, sites may share the fact-checking information or other sources to read, leaving it up to readers to assess their own biases and make a reasoned judgment about the likely truth of online information—a skill that is not widely taught nor mastered (Britt et al., 2019).

One method that avoids outright removal is to attach a warning tag to posts that have been disputed by independent fact-checkers. In response to criticism over the spread of doctored or misleading videos of 2020 presidential candidate Joe Biden, Twitter started broadening the use of “manipulated media” labels attached to posts sharing such photographs and videos, while still leaving them visible on the site (Lima, 2020).

However, while there is meta-analytic evidence that corrections (both forewarnings and rebuttals) to misinformation can be effective in some circumstances (Walter & Murphy, 2018), multiple studies on fake news have found limited effectiveness for social-media style warning tags (e.g., Ecker et al., 2010; Pennycook et al., 2018). One study found that warning tags using stronger language (saying an article has been “rated false” instead of just “disputed”) increased the effectiveness somewhat, but the impact was small, and the researchers did not look at belief over time (Clayton et al., 2019). Others have discussed the importance of giving a reason or alternative explanation for how the false claim came about (Lewandowsky et al., 2012).

### Sleeper effect

**Fig. 1:**
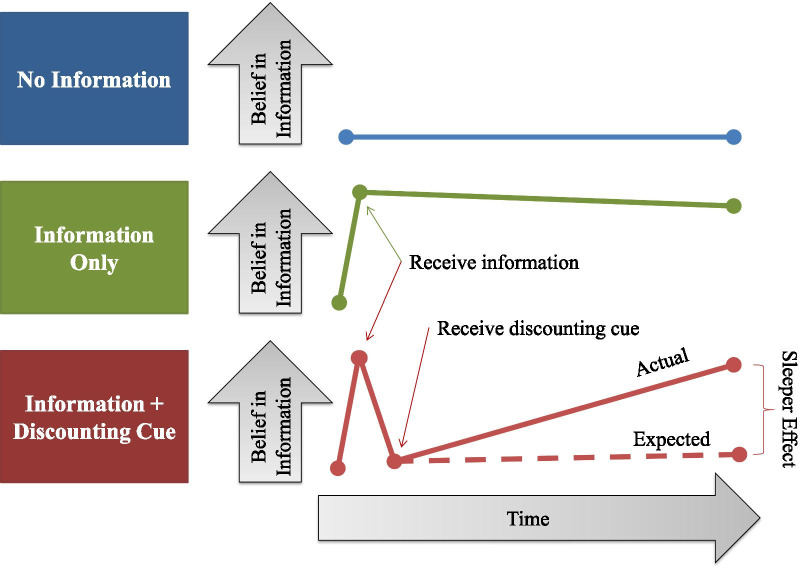
Visual explanation of the sleeper effect for three hypothetical groups of people. The top group receives no information so they never believe it, the second only receives the information so they believe it (with some fading), and the last receives the information plus a cue that tells them not to trust the original information. We would expect that after the cue, if trusted, people would look like the top group and continue not believing the information, but instead their belief rises over time, getting closer to the group that never received the discounting cue

Why might debunked fake news continue to exert influence over time? The “sleeper effect” occurs when there is an initial, persuasive message, followed by a new piece of credible information that discounts the initial message (e.g., a fake news warning label). Over time, the original message and the discounting cue become dissociated, such that the original message rises in persuasiveness in a person’s mind as its connection to the reason to disbelieve it weakens (Pratkanis et al., 1988). As the discounting cue, which makes a person disbelieve the information initially accepted as true, becomes dissociated from the message, the original information is once again treated as true when recalled later. This is why it is important for research on fake news warnings to look at impact over time; if viewers were to see the article again later without the tag (for example if a friend sent it to them or if they came across it on another platform), it would be important to know if the previous exposure to the “disputed” tag would allow them to recognize it as false, or if they would believe it once more. Figure 1 shows this effect visually—though the lines are abstract and illustrative, the basic pattern has been shown empirically (e.g., Pratkanis et al., 1988; Swire et al., 2017). We would expect those who receive a discounting cue to remain at zero belief over time, since they now know not to trust the information, but instead their belief in the original information increases over time.

Research on the sleeper effect may help explain why fact-checks often are not fully successful in correcting misinformation, as well as identify conditions when they are likely to be most effective (Swire & Ecker, 2018). Facebook, which has at various points attached labels below disputed articles to warn people to be wary of them, for a while stopped this practice because of the research showing its lack of or negative effect (Lyons, 2017; though used it along with contextual fact-checking articles to help combat COVID-19 misinformation; Clegg, 2020). A meta-analysis of the sleeper effect literature shows that there was no significant sleeper effect, meaning no rise in belief after a discounting cue, in studies where the discounting cue came *before* people heard the argument or information (Kumkale & Albarracín, 2004). In other words, if people were warned about the lack of credibility in information before receiving it, they processed it in a different way and persisted in *not* believing it over time. This fits with other research about correcting misinformation, in that forewarnings about upcoming misinformation are more effective than correcting the information after, though those warnings do not offer complete protection (Ecker et al., 2010; Loftus, 2005).

In response to growing research about the limited impact of warning tags below an article and backlash against the prevalence of false information on the platform, Facebook recently implemented a stronger warning, where the article image and headline is obscured by a warning that more clearly articulates that it was false information until users opt-in to viewing (Rosen et al., 2019). As this type of intervention is newer, and not fully rolled out, there is little direct, public research on how effective it may be compared to prior styles.

### Political motivation

The aforementioned studies suggest that specific, strong, prior warnings that come before fake news headlines are more likely to be effective than the warning label tags under an article that have been utilized widely in social media. However, these studies have mostly been in non-political realms, and the additional factor of political motivation may limit the effectiveness of prior warning, as political misinformation is harder to correct than other realms such as health information (Walter & Murphy, 2018). Misinformation is especially powerful when it supports a person’s worldview (Swire & Ecker, 2018), and warnings or retractions are especially likely to be ineffective if they are interpreted as an attack on a person’s identity (Paynter et al., 2019).

Pennycook et al. (2018) studied the effect of warning tags for politically oriented fake news headlines that were presented multiple times and found that participants were more likely to believe stories that were congruent with their political leaning (similar to other forms of biased information processing, seen in Ditto et al., 2018). A secondary analysis of their data (see full details in Additional file 1: Appendix A) shows some directional, but not statistically significant, indication that a warning tag reduced the effect of political congruency, in that the tag reduced belief particularly in politically congenial information. However, one week later, belief in politically friendly fake news was high once more.

### Purpose of the present study

While some prior studies have included longitudinal measures, most are conducted in single sessions, assessing initial reactions or behaviors in response to warning tags or other fake news interventions, and many looked only at warning tags below headlines like Facebook used to use. These immediate reactions are highly important to assess, but it is also crucial to look at belief over time, given what we know about the sleeper effect and the change in effectiveness over time seen in prior studies. This study addresses these areas by examining the effectiveness of various forms of fake news warnings or corrections, assessing how they interact with political congruency of the false information, and evaluating over a two-week time period to look at lasting impact of exposure to fake news and the effectiveness of potential tags.

The goal of this study is to see if we can improve fake news warning labels by taking what has been learned from the misinformation and sleeper effect literature and applying it to online political news through a warning that comes before a headline can be seen and has a strong message that the item is false. Additionally, we wanted to assess effectiveness over time to reflect the real-world scenario where people see fake news while browsing online and then are affected by it at a later date, for example when having a political conversation, deciding who to vote for, or judging a speech on TV. For a particular social media company looking to improve warnings, the first, immediate judgment may be the most important, as long as they can ensure the information is always tagged (and thus there is no repeat exposure without a warning). However, a piece of false information may be posted again by a new content provider, so having people be able to reject false information they see again later would be beneficial.

Our study used a paradigm where people see fake headlines within a group of true headlines and are warned about their inaccuracy. We included a condition where people were warned *before* the false headlines, similar to Facebook’s new false information warning, and compared this to the traditional label below a headline and to a correction that came after participants had read and judged the headline (which also allows a baseline measure of the belief in the news item without a label). We assessed belief in the false information both immediately and after a two week delay.

Our primary prediction was that giving people a warning before false information would be the most effective in promoting disbelief in information initially. If the pre-warning prompts people to think more deliberately or analytically about the headline as they read it (Sindermann et al., 2020), then they may also show less difference between fake news that supports or opposes their political allegiances (a reduced political congruency effect). Our core research question was how these warnings would fare over time. We expected that the warning before the headline would show the least sleeper effect, in that there would be less (or ideally no) rise in belief over time as compared to corrections that came after the headline.

## Method

### Sample

Participants were recruited from Amazon’s Mechanical Turk in February 2019. The study took place over two sessions, two weeks apart, and paid $0.75 for each 4–8 min sessions. Workers had to be US citizens, be over the age of 18, come from a US IP address, and agree to take part in both sessions. The first survey collected data from 541 individuals, and the second had 429, for a return rate of 79.3%. Importantly, the return rate did not differ based on political affiliation (*χ*^2^(2) = 4.265, *p* = 0.119) or on experimental condition (*χ*^2^(2) = 0.839, *p* = 0.657).

For many of the following analyses, only the 418 participants who fully completed both surveys were included. This ensures that the sample is consistent between analyses in order to make appropriate comparisons at each timepoint. Given the three experimental conditions, with 80% power and two-tailed α of 0.05, we can detect an *f* effect size from a one-way ANOVA as small as 0.152 (which is equivalent to a Cohen’s *d* of 0.305, a small-to-medium effect).

### Time 1 materials and procedure

After consent and completion of demographic questions, participants were asked a few questions about their political behavior, including their interest in following political news and what political party they identify with. Those who did not select either Democrat or Republican initially were asked which of the two they leaned toward (or none). Leaners were grouped with party identifiers for political affiliation, as is commonly done in political polls because independents who profess a leaning to one party generally vote and behave similar to party identifiers (Keith et al., 1986; Klar & Krupnikov, 2018). People were also given some initial questions about feelings toward political groups for exploratory moderator analyses discussed in Additional file 1: Appendix E.

#### Ratings of news headlines

In the main body of the survey, people saw a series of 15 news-like headlines, each presented on its own page under a photograph similar to the cards that would be seen on a social media feed (all cards can be found at https://osf.io/gtuha/). Below each headline were two questions asking participants how interesting the story was (from 1 = “Not at all interesting” to 5 = “Extremely interesting”) and how accurate they thought it was (from 1 = “Completely false” to 5 = “Completely true”).

Twelve of the headlines were created based on multiple credible sources from mainstream news and thus are considered “true.” Three of the headlines were made up by the first author and checked online to ensure there had not been any news stories on the topic (whether true or false), and were the “false” stories. To pick the false items used in the study, we conducted a small pilot on Reddit with a list of many made-up news headlines created by the first author and chose the ones that were most broadly believable to people across political parties (study details in Additional file 1: Appendix C) and were relatively matched in content type.

For both the true and false headlines there was an equal mix of headlines considered to be “Democrat-friendly,” “Republican-friendly,” and “Politically neutral,” with the partisan-friendly true news either being positive toward that party or politicians or negative toward the other party (false news headlines were only negative since there was only one of each persuasion). The headlines were randomly ordered for each person, except that the false headlines could not be one of the first three seen, and the first and last headline was always a politically neutral true item in order to not put people into a political or skeptical mindset from the start or leave them in one at the end. Table 1 shows all of the false headlines used, as well as one example of a true headline of each type. All study materials and headlines are in Additional file 1: Appendix B.

**Table 1:** Examples of true headlines and all false news headlines used in study

	False headlines (all)	True headlines (one example each)
Democrat-friendly	RNC Chair Ronna McDaniel called President Trump “f***ing idiot” in a closed meeting and suggested it may be better if Democrats win the next election	President Trump’s 2017 inaugural committee is said to be under criminal investigation by federal authorities due to financial fraud around donations
Republican-friendly	Discussing voter fraud allegations in private meeting, Tom Perez, DNC Chair, suggested that electing Democrats “more important” than the letter of the law	Online group fighting to outlaw alcohol found to be Democratic activist campaign designed to reduce support for Republican candidate Roy Moore
Politically neutral	Leaked company documents show top E-cigarette company Juul–which insisted it didn’t market to teens–sought teens for focus groups and as models	The Weather Channel is being sued over accusations that it is illegally collecting and selling user’s personal location data

#### Warning condition

**Fig. 2:**
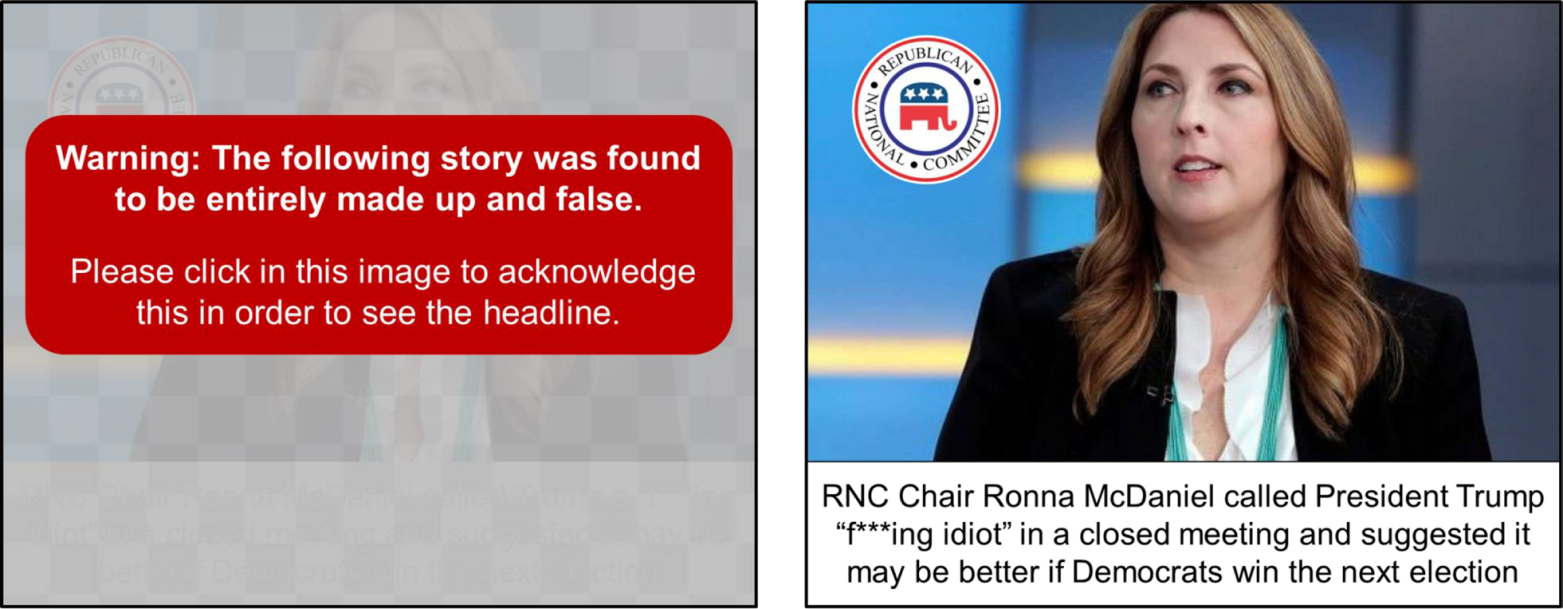
How the “Warning-Before” for a false headline appeared to participants before clicking (left) and after clicking (right). True headlines would be in a similar style to the card on the right, while “Warning-During” false headlines had a similar red box below the headline (with the text referring to the “above study”), and the Warning-After were given similar text on the next page about the “previous study”

For the three false news items, participants were randomized to one of three warning conditions and saw the same warning type for all three fake news items they rated. Those in the “Warning-After” condition were only told after they had answered the two questions about the article (its interestingness and accuracy) that the headline was false; when they advanced to the next page they were told, “Warning: the story on the previous page was found to be entirely made up and false.” Although this would be considered a “correction” and not a warning since it came after the information, we have kept the usage in regard to this condition throughout to match what was used with participants and to keep a consistent terminology between conditions. In the “Warning-During” condition, most similar to previous studies and the labels used on some social media sites, a box with similar language (saying the “above” story instead of “on the previous page”) was presented directly under the article as part of the image. Finally, in the “Warning-Before” condition, the headline and image were obscured and covered with a box containing a similar warning about the “following” image, similar to Facebook’s recent label warning. Participants had to click to acknowledge they understood that before they could see the image and answer the questions; see Fig. 2 for an example.

Participants saw the same warning for all three news items in their survey, and there was no condition where people were not told that the items were false. However, since people in the Warning-After condition rated the accuracy judgment of the items before the warning, this is a control measure of the baseline belief in the fake news item for comparison with other conditions, at least at Time 1.

**Fig. 3:**
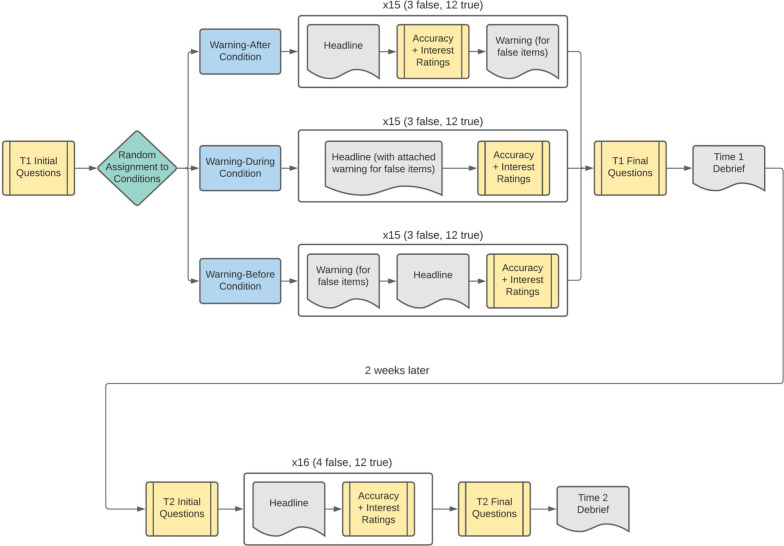
Study procedures diagram

At the end of the first survey, participants were reminded that they saw both true and false headlines and were told that all the ones they were told were false really were made-up. They were reminded about the follow-up in two weeks and given a chance to leave feedback in an open-ended text box. A flowchart of the procedure for each condition is shown in Fig. 3.

### Time 2 materials and procedure

Two weeks after they completed the first survey, participants were emailed a link to complete the second survey, with a reminder email three days later to those who had not completed yet. The Time 2 survey was open for a total of seven days to ensure that participants had enough time to take the survey, but not so long that they could have a much longer time period between surveys than other participants. This is a longer interval than some similar studies of misinformation that often use a one-week interval, though it is similar to the mean interval of 18 days used in past sleeper effect literature (Kumkale & Albarracín, 2004).

The Time 2 survey was similar to the first, starting with two 5-point self-report questions about how much participants had used social media and how much they had followed political news since the prior survey. Next, in a similar format to the Time 1 survey, they again saw a series of headlines with images above them and questions below them. All three of the false headlines were presented again along with nine of the previous true headlines (leaving out one Democrat-friendly, one Republican-friendly, and one politically neutral true news item). Additionally, there were four new headlines added that were drawn from recent news: one true Democrat-friendly, one true Republican-friendly, and one true politically neutral news item, as well as one politically neutral fake news items that was taken from Snopes.com. There were no warnings during the headline rating phase in the Time 2 survey, as the goal was to assess the long-term effectiveness of the original warnings, so all participants had a similar experience regardless of initial condition (see Fig. 3).

Under each headline participants were again asked to rate how interesting and accurate the story was on a 5-point scale. In addition, they were asked if they remember seeing the story in the Time 1 survey two weeks ago (Yes, Unsure, or No) and if they had seen anything about this story outside of this survey (Yes, Unsure, or No).

#### Memory bias awareness

There were two more sections at the end of the survey after the headlines. One section asked about their awareness of their own potential biases, with questions about how accurate they thought they had been, which type of headlines (politically congruent or politically incongruent) they were more likely to search for more information about, how effective they thought the warnings they had seen would be, and how good they generally are at spotting fake news. The other section was a page that presented a list of all 19 headlines seen across both surveys, and participants were asked to decide whether each was—on the whole—more likely “True” or “False.” Participants were randomly assigned to receive either the self-awareness questions first or the True/False judgment first. This was for an exploratory investigation of whether prompting people to think about their biases, which may put them in a more critical thinking state, would affect their accuracy or skepticism in judging the news items in a final measure (presented in Additional file 1: Appendix E).

#### Debrief

At the end of the second survey, participants were reminded which headlines were false and were told the false headlines were made up for the study. They were then allowed to write any comments or thoughts before being thanked and paid for the second session.

### Measures

#### Outcome variables

The main outcome was the judgment of the accuracy (on a scale of 1–5) of each of the false news items rated at both Time 1 and Time 2. Ideally, all would be a “1” because the items were all false, so anything above that indicates some belief in the information. Additionally, participants made a binary judgment at the end of the Time 2 survey regarding whether each of the three fake news items (and each of the true items) was true or false. This was used to get a count of how many of the three false items they (incorrectly) judged to be true at the end of the study.

#### Predictor variables

The first main predictor of interest is experimental condition, a three-level categorical variable: Warning-Before condition (*n* = 139), Warning-During (*n* = 136) condition, and Warning-After condition (*n* = 140). In regressions the Warning-Before was chosen as a reference group in order to compare this novel version to both the most likely to be least effective condition as well as to the common current practice of warnings below headlines.

The second main predictor is whether the headline being judged was congruent or incongruent with a person’s political beliefs. For example, for a Democrat participant the Democrat-friendly news items would be “congruent” and the Republican-friendly news “incongruent,” and vice versa for a Republican participant. Including those who leaned toward either party there were 255 Democrats and 110 Republicans for this analysis (leaving out the 50 non-partisans for this question).

Other predictors used for fake news belief and supplemental moderator analyses include social media usage (a 5-point self-report question at Time 2 about the past two weeks), the number of true headlines correctly rated as true (which may indicate a general response bias toward “true” or may indicate increased knowledge of political news; O’Connell & Greene, 2017), conspiratorial thinking (measured by a single 5-point item about agreeing with the statement, “Big events like wars, recessions, and the outcomes of elections are controlled by small groups of people who are working in secret against the rest of us” taken from Uscinski et al., 2016), and interest in political news (a 5-point self-report question). These will help assess personal characteristics that may be related to a generally increased belief in fake news items. Descriptives of these and other relevant variables are given in Table 2.

**Table 2:** Various descriptives and demographics of main completer sample

	*M* (SD)
*Personal variables*	
Conservatism (1 = “Extremely Liberal” to 7 = “Extremely conservative”)	3.32 (1.69)
Belief in global conspiracies (1 = “Strongly disagree” to 5 = “Strongly agree”)	2.63 (1.28)
Interest in political news (1 = “Not interested at all” to 5 = “Extremely interested”)	3.47 (1.03)
Political party (1 = “Strong Democrat” to 7 = “Strong Republican”)	3.21 (1.99)
Social media use between surveys (1 = “Not at all” to 5 = “A great deal”)	3.24 (1.09)
Intention to vote in next election (1 = “Extremely Unlikely” to 5 = “Extremely Likely”)	4.47 (1.04)
*Feelings toward political institutions*	
Feeling toward DNC (0 = “Completely cold/negative” to 100 = “Completely warm/positive”)	44.41 (29.31)
Feeling toward RNC (0 = “Completely cold/negative” to 100 = “Completely warm/positive”)	29.42 (28.36)
*Trust in information (1* = *“Not at all” to 5* = *“A great deal”)*	
Trust in information from online news sources	2.74 (0.92)
Trust in information from social media	2.21 (1.01)
Trust in information from traditional news sources	2.90 (1.08)
Trust in information from government resources	2.60 (1.07)
Trust in information from family and friends	2.65 (0.96)
*Demographics*	%
Democrat	54%
Republican	26%
Non-leaning independent	12%
Male	55%
Female	44%
Other/non-binary	1%
White	78%
Black/African–American	6%
Asian–American	6%
Multi-racial	5%
All others	4%

Since there are more Democrats in the sample than Republicans, the overall averages lean more liberal/Democratic, though many analyses are agnostic of this, and we did not find party differences in the main outcomes

## Results

### Accuracy judgments

**Fig. 4:**
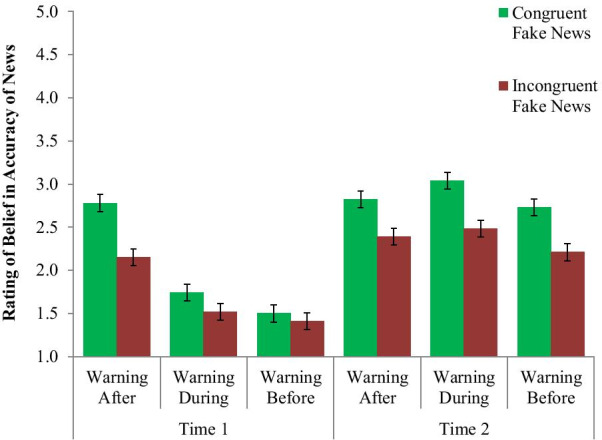
Partisan participants’ ratings of the accuracy of politically congruent and incongruent fake news headlines at Time 1 and Time 2 based on warning condition. Error bars indicate one standard error of the mean

The main outcome was the rating of the accuracy of the news items depending on the warning condition (Before, During, or After headline and rating), political congruency (politically friendly or unfriendly news), and time (Time 1 or Time 2). The results for each group are shown in Fig. 4. From this graph, we can see that at Time 1, people in both the Warning-During, and especially in the Warning-Before condition, appear to believe the false news items less than in the Warning-After condition (where they judged the articles before the warning), with much smaller differences between politically congruent and incongruent news. At Time 2 however, the conditions all appear much more similar, with moderate levels of belief and a sizable difference between congruent and incongruent news. Thus, it appears that the partisan congruency effect was reduced with the warnings but returned over time.

We conducted a number of statistical analyses to test the patterns that appear in the results. The primary analysis was 3-way linear mixed regression, predicting accuracy judgment of political fake news items based on warning condition, congruency with participant’s political views, time, and their interactions. The analysis controlled for within-person variation in general believability by adding subject ID as a random effect, allowing each person to have their own intercept. We used only random intercepts, not random slopes, in order to keep all the models a similar structure across different analyses (even ones with differing variables or that did not support the extra complexity). This analysis only considered the Democrat-friendly and Republican-friendly news items and only included those who identified or leaned toward one of those parties (*n* = 365, 87.3% of the sample) in order to create the congruency value (which would be missing for the politically neutral items and participants). The Warning-Before variable was used as the reference group in the dummy coded condition variable to compare it to both the Warning-During and Warning-After condition.

This analysis found a significant 3-way interaction between warning condition, political congruency, and time for the Warning-After condition (*b* = − 0.618, SE = 0.244, *p* = 0.011), but not for Warning-During (*b* = − 0.077, SE = 0.244, *p* = 0.752). Looking at Fig. 4 and the interaction terms, we can see that the amount that the Warning-Before reduced the impact of congruency on fake news accuracy ratings, relative to the Warning-After condition, reduced over time, but that this change in congruency impact over time was not significantly different between Warning-Before and Warning-During. To follow-up on this interaction, we conducted separate two-way linear regressions (warning type x political congruency) at each time point.

At Time 1, there was a significant interaction between Warning-After and congruency (*b* = 0.549, SE = 0.161, *p* < 0.001) but not Warning-During and congruency (*b* = 0.139, SE = 0.161, *p* = 0.389). Running the regression for warning condition under each level congruency showed that the people in the Warning-Before condition believed the false news items less than those the Warning-After condition for both the congruent (*b* = 1.283, SE = 0.149, *p* < 0.001) and incongruent (*b* = 0.734, SE = 0.120, *p* < 0.001) items, while the interaction shows that the former was especially pronounced (i.e., the Warning-Before brought down belief in the congruent false news even more).

When looking at the impact of congruency on Time 1 ratings in separate regressions for each condition, congruency had a significant impact on belief in fake news for those in the Warning-After condition (*b* = 0.631, SE = 0.150, *p* < 0.001), but not one for those in the Warning-During (*b* = 0.221, SE = 0.134, *p* = 0.104) or Warning-Before (*b* = 0.0832, SE = 0.120, *p* = 0.492). A 2-way analysis (with Warning-After as the reference level) showed that both the Warning-During (*b* = − 0.410, SE = 0.160, *p* = 0.011) and Warning-Before conditions (*b* = − 0.549, SE = 0.161, *p* = 0.001) had significantly smaller effects for the congruency variable, meaning less difference in belief between congruent and incongruent fake news. This means that both warning types were successful at suppressing the impact of political congruency (down to a non-significant amount), and that while the Warning-Before condition showed the lowest congruency impact, it was not significantly different from Warning-During.

At Time 2, however, there was no such two-way interaction between warning condition and congruency for either the Warning-After (*b* = − 0.070, SE = 0.170, *p* = 0.638) or Warning-During (*b* = 0.061, SE = 0.170, *p* = 0.719), meaning the effect of the Warning-Before in reducing the impact of congruency had gone away. Looking just at the main effect of warning condition and congruency (without their interaction) at this timepoint, we find a significant main effect of congruency (*b* = 0.501, SE = 0.069, *p* < 0.001). For warning condition, we find that the Warning-Before condition was not significantly different from the Warning-After condition (*b* = 0.136, SE = 0.116, *p* = 0.244), but was a bit better (in terms of having lower fake news belief) than the Warning-During condition (*b* = 0.291, SE = 0.116, *p* = 0.013).

Overall, and as shown in Fig. 4, this indicates that the Warning-Before showed an immediate (Time 1) impact relative to the Warning-After in reducing belief in the fake news articles and eliminating any significant impact of political congruency, but was not significantly more impactful compared to Warning-During. Over time, however, that impact lessened, to where the Warning-Before was no better than either other condition in reducing the impact of political congruency. It did, however, perform a small amount better than the Warning-During condition at Time 2 in lowering belief on average. Analysis of each headline individually is in the online supplement, Additional file 1: Appendix E, along with tests of various individual difference moderators of the effect, and the raw output of all models can be found at https://osf.io/gtuha/.

**Fig. 5:**
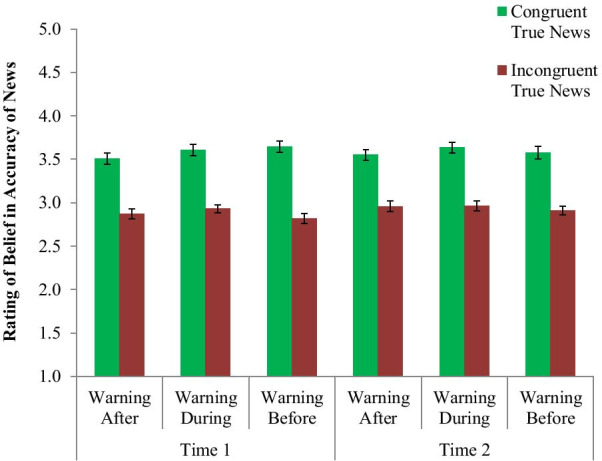
Partisan participants’ ratings of the accuracy of politically congruent and incongruent true news headlines at Time 1 and Time 2 based on warning condition. Error bars indicate one standard error of the mean

As a comparison, the ratings of true news articles showed no such impact of warning condition or time, as seen in Fig. 5. When running the same analyses above, except using the rating of true articles (averaged across multiple items for each participant), there were no significant two- or three-way interactions between warning condition, congruency, and time. The only significant effect was a main effect (when running a linear mixed regression with those variables, without interactions) of political congruency (*b*** = **0.679, SE = 0.030, *p* < 0.001) the impact of which did not change based on timepoint or warning condition. This means that people believed the true news more when it was favorable to their political views, and neither this effect nor overall belief changed over time or depending on warning condition.

### Memory of headlines

**Fig. 6:**
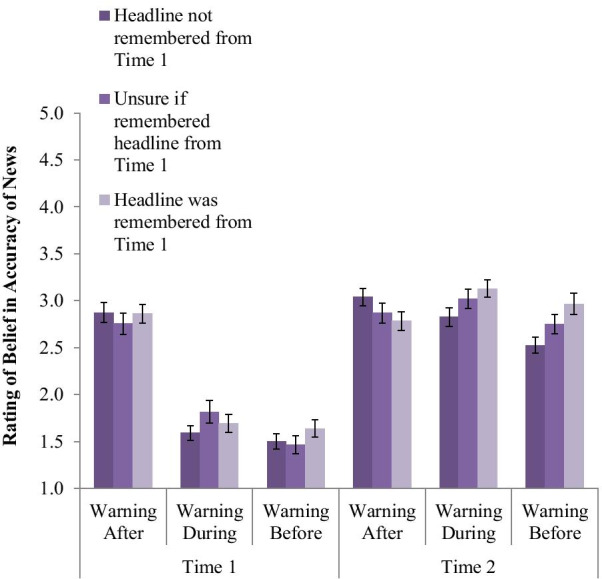
Belief in fake news accuracy by warning condition, time, and how confident the participant was in remembering, at Time 2, that they had seen that headline previously in the Time 1 survey

We also looked at the impact of whether participants reported that they remembered seeing the headline at time 1 by running a similar 3-way mixed regression predicting belief in the fake news item by warning condition, time, and memory of seeing the headline (treating the three-point measure as a scale, with higher numbers being more confident of memory). This analysis was done on the full sample of respondents and false news items, as it did not include the congruency variable that required limiting to non-partisans. On average across the false news items, 36% of respondents reported that they did not remember seeing the headline in the survey at time 1, 24% were unsure if they had seen it, and 40% remembered seeing it. There was statistically significant a 3-way interaction such that the relationship between memory and accuracy changed over time differently between the Warning-Before condition and Warning-after condition (*b* = − 0.282, SE = 0.121, *p* = 0.020), though there was no significant difference between Warning-Before and Warning-During (*b* = − 0.052, SE = 0.120, *p* = 0.666). Follow-up analyses show that at Time 2, those in the Warning-Before condition believed the fake news more the more they remembered it from Time 1 (*b* = 0.249, SE = 0.069, *p* < 0.001), whereas there was no significant impact of this memory variable in the Warning-After condition (*b* = − 0.110, SE = 0.068, *p* = 0.108), as seen in Fig. 6. And there was no significant impact at Time 1 based on whether that headline was eventually recalled at Time 2 or not (*b* = 0.060, SE = 0.038, *p* = 0.110). The lowest belief in the fake news headlines at Time 2 came from people who saw the Warning-Before but did not remember seeing it at Time 1.

For the question about whether people remembered seeing the false story anywhere outside of the survey (which should not have happened since they were created for the study), 20% of participants reported remembering at least one of them. This rate did not differ between warning condition (χ^2^(2) = 0.458, *p* = 0.795) and did not impact warning effectiveness between any conditions or their interactions over time (all *p*s > 0.05). It did, however, interact with the time variable (*b* = 1.032, SE = 0.245, *p* < 0.001), in that those that reported remembering the story outside of the survey rose significantly more in their belief from Time 1 to Time 2.

### Count of false items believed

Finally, we conducted a regression predicting the number of the false items rated as “True” in the final assessment of all news items to find other possible individual factors (e.g., political disposition, trust in online news) that may increase susceptibility to false news items. This analysis used a Poisson regression (overdispersion estimate = 0.823, *p* = 0.996, indicating overdispersion was not an issue) on the full sample. Coefficients of the model are in Table 3.

**Table 3:** Predictors of increased number of false items believed to be true

	Poisson regression model predicting count of false items rated as true at end		
	Coefficient	Standard error	*p* value
*Warning condition*			
Warning-After	0.128	0.108	0.238
Warning-During	0.087	0.110	0.428
*Order condition*			
Memory items first	0.086	0.087	0.322
*Personal variables*			
Male gender (relative to female)	0.031	0.090	0.732
Ideology (higher is more conservative)	− 0.010	0.027	0.718
Interest in politics	− 0.034	0.044	0.434
Conspiratorial disposition**	0.108	0.035	0.002
*Online news variables*			
Social media usage	0.015	0.043	0.717
Trust in social media	− 0.015	0.051	0.765
Trust in online news	− 0.021	0.054	0.702
True news stories rated as true***	0.074	0.022	0.001
Constant	− 0.500	0.312	0.109

Items with *** were statistically significant at *p* < 0.001, ** are significant at *p* < 0.01, and others were not significant (*p* > 0.05). The reference categories were Warning Before for Warning condition, Bias awareness questions first for Order condition, and female for Gender (with other genders excluded due to sample size)

On average, participants believed 1.36 of the fake news items (out of 3) at the end. There was no impact of either the warning condition or the order of whether the respondents first rated the items as true or false or first gave an assessment of their own potential bias. For the personal variables, there was no significant effect of gender, political ideology or being more interested in politics. However, there was a significant effect of conspiratorial belief, measured by how much participants agreed or disagreed (on a single 5-point item) that big events are controlled by a secret group working against the rest of us. Importantly, conspiratorial thinking was only predictive of believing more false stories; there was no significant correlation between that conspiracy item and believing more of the true stories (*r* = − 0.030, *p* = 0.544).

Finally, the model included self-reported social media usage in the prior two weeks, self-reported trust in online news sources, and self-reported trust in information found on social media, none of which predicted the number of false items believed as true. We did find that the more true news stories were (correctly) believed as true, the more false items were also rated as true. A correlation table between items is available in Additional file 1: Appendix D.

## Discussion

The warning that came before the headline had a strong impact on getting people to reject the fake news and getting them to show no difference based on the political congruency of the information. However, its improvement over the more common label under the headline was not statistically significant at that time. And over time, that difference from the correction condition, the closest we had to a control condition, largely went away. Two weeks after reading the headline, people who had received that strong warning rated the accuracy of those news items at similar levels as those who had received the warning after the headline, meaning the warning did not have an “inoculation” effect as hoped, where the news would be remembered as false if it came up again elsewhere. This mostly contradicts our expectation based on prior sleeper effect literature, which has shown a lack of a sleeper effect when the discounting cue came before the information (Kumkale & Albarracín, 2004), though it did have a slight improvement compared to the concurrent warning label. By Time 2, the effect of political congruency strongly returned and at similar levels in all conditions. This shows that even after a strong, initially effective warning, what remains is the on-going tendency to believe what fits with our political views. As memory fades and warning effectiveness lessens, the impact of partisanship stays on after a temporary setback—partisanship persisted.

It is possible that the warning before may have shown more of a positive impact one week out (if the before warning decayed slower than others), though past analyses have not found a significant impact of time interval on the sleeper effect (Kumkale & Albarracín, 2004). Future studies may look at varying the time interval, as people may be re-exposed to misinformation in the real world anywhere from minutes to years from when they first saw it.

There was a mix of people who remembered the headline from the prior survey and people who were unsure or did not. When looking at belief at Time 2, those who were in the Warning-Before (the forewarning condition) were better at rejecting the fake news headlines the *less* they reported a memory of that headline from the Time 1 survey. Other research has found that feeling more confident in one’s memory can be associated with more biased recall of thinking that past political attitudes were more similar to current attitudes than they actually were (Grady, 2019); it could be that the feeling of familiarity of a story that leads to more reported (but not actual) memory also leads to reduced effectiveness of the otherwise strong warning because that familiarity is also associated with truthfulness (Polage, 2012; Whittlesea, 1993). Future studies may want to investigate not just whether people remember seeing the headline before but whether they remember seeing the disputed notice on it, since the memory of the information alone may have been encoded as true simply by reading it, even after the warning (Gilbert et al., 1993). About 1/5th of the sample reported a potential false memory, reporting remembering at least one of the stories from outside of the survey, and those who did were especially likely to have come to believe the story was true later.

As expected, political congruency was highly related to the final accuracy judgments, such that people thought that headlines that were more friendly to their political affiliation were more likely to be accurate than news that was not, even though participants had been told that all of them were false and had shown less of a congruency affect with a warning at time 1. Past research has shown that this political effect is driven not just by motivation to find truth in the friendly story, but that the congruent information is accepted more uncritically or “lazily,” while the incongruent information is given more critical thought about why one should be (rightfully) skeptical of it (Ditto & Lopez, 1992; Pennycook & Rand, 2018).

This effect occurred similarly for both Democrats and Republicans, who did not significantly differ in the amount they were affected by political congruency of the news nor in how much they believed false news on average. This is not surprising given past research showing similar levels of information processing bias between liberals and conservatives (Ditto et al, 2018). This difference was present for the true news items as well, which were steady over time and across conditions, indicating that the presence of these various types of warnings did not significantly impact how people rated headlines that they saw without any warnings.

These results do not suggest that warnings have no benefit or that the new Warning-Before model is not a better choice than the other versions like the concurrent label or post-correction. While it did not show as strong of a long-term effect as hoped, where most people would continue rating the false news as false multiple weeks later, the before-warning was still the best overall. It had a strong initial impact relative to the correction condition, and a slight long-term advantage over the more commonly used concurrent warning (though not significantly better than the correction condition expected to have the worst long-term performance), making it the same or better than the other version at every test.

Importantly, this study required people to view all the headlines in order to assess memory for them at follow-up. However, when given a forewarning and given an option to not view, it is possible that many users would opt to avoid reading the article in the first place, whereas those with the warning label next to an already-visible headline may not have that option to choose to not expose themselves to the false information. Thus, the Warning-Before may be a much more powerful warning in real-world usage if it causes people to avoid the information entirely, something not assessed in this study but worth future investigation.

One individual variable that did come out as a significant predictor of believing false headlines in general was conspiratorial thinking, which matches with prior research showing those higher in conspiratorial thinking are more susceptible to believing in politically oriented false news (Anthony & Moulding, 2019; Grady, 2019). It may be that this goes along with a less analytical style or more dogmatic form of thinking that other researchers have found associated with belief in fake news (Bronstein et al., 2018), and that targeting specific groups of people or trying to increase analytical thinking style may be important in the future. Those high in conspiratorial thinking are more likely to share fake news due to their increased belief in them (Halpern et al., 2019), so any reduction in their long-term confidence is likely to limit sharing behavior as well.

Given these noticeable but modest (in the long-term) impacts, these results suggest that warnings are not likely, alone, to be sufficient to counter the impact of false information online, given how hard misinformation is to correct once read. Even if people recognize an article as false the first time they encounter it, they may believe it just as much if seen again later without a tag. Additional measures should also be trying to limit the amount of false information that is distributed in the first place, rather than trying to relying only on warnings or corrections to mitigate their impact, such as how various sites like Facebook and YouTube are now starting to remove content that aims to misinform people.

### Limitations

There were several important limitations of this study. First, people knew they were participating in a research study. This may have caused the warnings to be less effective because people were suspicious of the agenda of the researchers in giving the warning, or the warnings may have been more effective than they would be in the real world if people trusted in the authority of the information (e.g., one participant commented that they wished to know *who* had rated the story as false, as that would help them decide whether to trust it). The act of asking participants to rate articles makes them more critical in judging accuracy (Moravec et al., 2019), meaning that real-world belief is likely even higher. Future studies should look to real-world settings and behavior (e.g., opting in to view articles, sharing behavior) to understand how these warnings work in appropriate contexts.

Power was adequate for the between-subjects test for a medium size effect, but given the modest impacts of warnings found in other studies, and the many subgroups to test, a larger sample might be needed to detect smaller effect sizes, especially between the Warning-Before and Warning-During conditions.

The design of the study used three fake news items, and many analyses focused on the two partisan items. If there were issues with the believability or reception of a particular item, that may impact the results in a way that a larger study with many items may be able to balance out. We chose to use just one of each type to keep them balanced to each other on believability and consistent across participants, but future replications with larger, more diverse set of fake news items (varying in topic and believability) would improve our understanding of the generalizability of the results. For example, the politically neutral fake news item was believed more often because it was more similar to actual accusations that had happened against the company. This presents an important aspect for future research to address, in that often the “fake” news being spread is not 100% false, instead but misleading or incorrect in some way. News that is similar to real events, or based on outdated information, or presented in a misleading manner may have similar negative effects as a completely untrue story, but may be harder to detect and thus correct than something entirely made up.

Finally, we did not have a true control group without warning or correction at time 1 to know whether belief would have risen over time regardless. This concern is lessened by seeing that true items (which had no warnings) did not show any increase over time, and by the fact that we were mostly comparing warnings to each other and not a control. We made the choice not to have to condition for ethical considerations, wanting to avoid a situation where we gave any participants negative information about two current presidential candidates that we left uncorrected for two weeks (and not corrected at all for those who did not return).

### Conclusions and future directions

These results are in line with other research showing the limits of effectiveness in warnings and the high, lasting impact of political congruency. This is important as social media organizations and other online sites continue to try and rely upon fake news warning labels, which may not be effective alone at combating the spread of misinformation. Overall, we found that the warning before a headline is likely an improvement upon the warning below a headline, but with a disappointing long-term effectiveness that warrants it not being the only solution.

Research on the continued effects of misinformation on memory have pointed out that the reason corrections are often ineffective is that the original information has already been accepted as legitimate and true (Seifert, 2002), and corrections are least effective when the information fits strongly with a person’s worldview (Cook et al., 2015). As negative affect toward opposing political parties increases (Iyengar et al., 2012), negative information about opposing candidates and their immoral behavior is likely to be accepted readily by partisans, even when in a skeptical mindset. In a practical sense, this shows how difficult it is to encourage rejection of politically congenial fake news; the news that people want to believe is likely to be accepted over time, and the rest rejected, leading to a self-fulfilling cycle of partisan expectations.

Given this, it may be that headline-level interventions are not going to be completely effective, and structural changes that limit the spread of fake news in the first place are needed to more effectively contain its dangerous influence (Levi, 2017). While any change to increase the effectiveness of warning tags would be beneficial, suggesting that a move to warnings before an article may be warranted, they need to be part of a broader solution, especially if people would not recognize the information is false if they encounter it again elsewhere.

Additionally, future interventions may want to look into addressing the emotional aspect in additional to the cognitive. Factual warning labels are a form of “cold” intervention—more cognitive, grounded, interventions designed to increase critical thinking—attempting to address a “hot” issue, where partisan emotions overwhelm people’s typical rational patterns. Seeing an outrage-inducing headline about a divisive political figure or topic feeds into increasing feelings of mistrust and anger (beyond just disagreement) toward the “other side” of the political aisle. While a person may immediately understand cognitively that a fact is false, it may be harder to undo the deeply partisan resonance that is felt when it fits well into an existing worldview.

## Supplementary Information

**Additional file 1.** Contains Appendix A (secondary data analysis), Appendix B (all study materials), Appendix C (pilot study to select materials), Appendix D (correlation table for individual variables) and Appendix E (other analyses not included in main paper).

## Data Availability

All study materials are available in Additional file 1: Appendix B. Data and scripts used to generate the findings in this paper are available from the first author by request, with some materials, models, and output available at https://osf.io/gtuha/.
