# Supplementary material for: Nevertheless, partisanship persisted: fake news warnings help briefly, but bias returns with time
Source: Cogn Res Princ Implic. 2021 Jul 23;6:52. doi: 10.1186/s41235-021-00315-z (PMC8299168; doi:10.1186/s41235-021-00315-z)
Supplement: Supplementary file 1 — Additional file 1. Contains Appendix A (secondary data analysis), Appendix B (all study materials), Appendix C (pilot study to select materials), Appendix D (correlation table for individual variables) and Appendix E (other analyses not included in main paper). [file 41235_2021_315_MOESM1_ESM.docx]

Additional file 1

Table of Contents

[Appendix A – Secondary Data Analysis 2](#_Toc67215877)

[Appendix B – All Study Materials 5](#_Toc67215878)

[Appendix C – Pilot Study to select items 17](#_Toc67215879)

[Appendix D – Correlation Table for individual variables 19](#_Toc67215880)

[Appendix E – Other analysis not included in main paper. 20](#_Toc67215881)

# Appendix A – Secondary Data Analysis

The authors undertook a secondary analysis of data provided by Pennycook, Cannon, and Rand (2018) to look at the interaction of warning presences, ideological congruency, and time on belief in false news items presented more than once. Based on prior research, we hypothesized that the presence of a warning tag would reduce the effect of ideological congruency at the initial timepoint, but that the effect would return at a later time. In other words, when participants are told a story is false and then are immediately asked to rate its accuracy, most will rate the story as false regardless of whether it is politically congruent if the warning is generally effective right away. Over time however, as the effect of the warning wears off (which they found), people would especially raise their belief in the congruent story because it would have seemed more plausible and positive to believe before the warning, while the belief in the incongruent stories would remain low. Thus, we expected to see a significant three-way interaction between warning type, timepoint, and ideological congruency. More specifically, we expected that at Time 1 the warning would reduce the effect of political congruency (showing less or no difference in ratings between politically congruent and incongruent information), while there would be no such interaction at Time 2 (with a significant effect of congruency regardless of original warning type). This is because of a greater motivation to believe the news before the correction came, thus a stronger initial encoding that would show a stronger sleeper effect. We conducted a secondary data analysis of their paper to look for this interaction to help guide the development of this study.

**Secondary Data Analysis**

We used Pennycook et al.’s data posted to https://osf.io/txf46/ and corresponded with the first author to ensure we were using the proper variables. We collapsed the data across their other manipulations of whether items were presented in a testing phase or not and used the average accuracy rating of news items (separated by congruent or incongruent for each person) that were presented at both Time 1 and Time 2 (i.e., ignoring items presented in only one of those surveys). We conducted a 2 x 2 x 2 mixed ANOVA testing for the interaction between warning type (between-subjects; present or not present), political congruency (within-subjects; congruent or incongruent), and time point (within-subjects; Time 1 or Time 2) on the rating of the accuracy of fake news items. Results by warning condition are in Figure A.1 below.


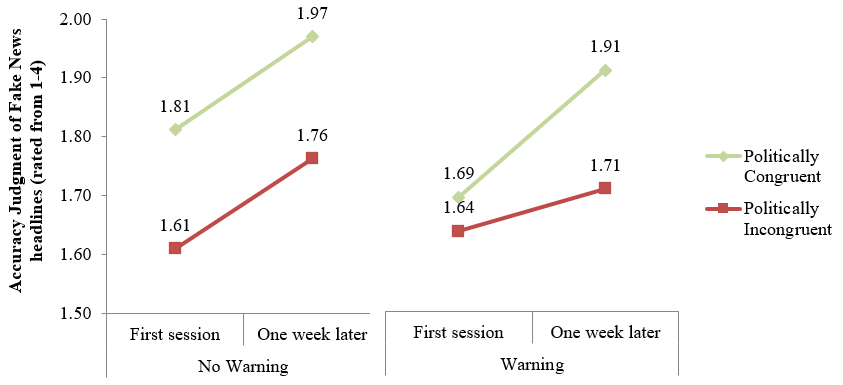


*Figure A.1*. Warning label on fake news only reduces belief in politically congruent fake news and only at initial timepoint.

The results are in the expected direction, but did not reach statistical significance for the three-way interaction, *F*(1,503) = 1.734, *p* = .189, likely due to the small effect of the warning at all which requires more power to detect. For example, at Time 1, there was the hypothesized two-way interaction when looking at the full sample (*F*[1,839] = 5.316, *p* = .021) in that politically congruent items were only rated as more accurate than incongruent items when there was not a warning. But when restricting it to the smaller sample of those who returned for the second survey, the same interaction was no longer statistically significant (*F*[1,504] = 3.444, *p* = .064) despite virtually identical mean values.

Looking at the values in each condition (see Figure A.1) suggests that the modest effect of the warning Pennycook et al. found at their first session was mostly due to lowering the accuracy rating of the politically *congruent* fake news item. In other words, people were already more skeptical of the politically incongruent fake news and the warning didn’t reduce that any further, while the warnings did make people more skeptical about the politically congruent fake news. While this was the case directly after the warnings were presented (although not statistically significant), within just one week, there was no difference between those who had and had not received a warning.

Though the evidence requires additional confirmation due to lack of statistical significance, the pattern supports the hypothesis that the motivation to believe the congruent fake news might make it especially susceptible to the sleeper effect, in that the content of the information would be more believed from the start and the false tag less strong (relative to the incongruent information).

# Appendix B – All Study Materials

Instructions:

This study will take place in two sessions about two weeks apart. In this first session, you will be reading a series of news headlines and answering questions about what you think of each one. You will also be asked some questions about yourself, such as demographics, political affiliations, and perceptions of news sources. The second survey will arrive in about two weeks from now.

We expect this to take approximately 10 minutes, with some variation. Please only complete this survey if you are over 18 years of age, in the United States, and willing and able to complete both HITs. At the end of the survey, you'll be given a randomized code that you will submit on Mturk to receive the payment.

Are you a U.S. Citizen?

o Yes

o No

What is your age in years?

________________________________________________________________

Are you willing to commit to taking a follow-up survey in two weeks?

o Yes, I agree to take the follow-up survey.

o No, I would like to take just this survey.

First we have some general questions about you.

What is your gender?

o Male

o Female

o ________________________________________________

What is your racial or ethnic identify? Check all that apply.

▢ White

▢ Black or African American

▢ American Indian or Alaska Native

▢ Asian or Asian American

▢ Native Hawaiian or Pacific Islander

▢ Middle Eastern or North African

▢ Hispanic or Latino/Latina

▢ Other: ________________________________________________

How interested are you in following political news?

o Not interested at all

o Slightly interested

o Moderately interested

o Very interested

o Extremely interested

Generally speaking, how you rate your ideological orientation, from extremely liberal (1) to extremely conservative (7)?

o 1 - Extremely liberal

o 2 - Liberal

o 3 - Somewhat liberal

o 4 - Moderate/Middle of the road

o 5 - Somewhat conservative

o 6 - Conservative

o 7 - Extremely conservative

Generally speaking, do you usually think of yourself as a Republican, a Democrat, an Independent, or something else?

o Democrat

o Republican

o Independent

o Other (please specify): ________________________________________________

o No preference

[If Republican] Would you call yourself a strong Republican or a not very strong Republican?

o Strong

o Not very strong

[If Democrat] Would you call yourself a strong Democrat or a not very strong Democrat?

o Strong

o Not very strong

[If Neither] Out of the following two, would you say you lean more towards the Democratic party or the Republican party?

o Lean Democratic

o Lean Republican

o Don't lean towards either

**Trust in groups**

We would like to know your feelings towards the following people and groups on a scale from 0 to 100. A score of 0 indicates a very cold/unfavorable view of the person or group, while a score of 100 would indicate a very warm/favorable view.

Cold/Unfavorable Warm/Favorable

0 10 20 30 40 50 60 70 80 90 100

Democrat voters

Republican voters

President Donald Trump

The Democratic National Committee (DNC)

The Republican National Committee (RNC)

Some people vote on national elections and some do not. Thinking about the next national election in 2020, how likely are you to vote in it?

o Extremely unlikely

o Somewhat unlikely

o Neither likely nor unlikely

o Somewhat likely

o Extremely likely

How much do you trust information that you receive from the following sources?

Never Sometimes About half the time Most of the time Always

Traditional news outlets o o o o o

Social media o o o o o

Online-only news sources o o o o o

Government information o o o o o

Friends and family o o o o o

Please rate how much you agree with this statement: "Big events like wars, recessions, and the outcomes of elections are controlled by small groups of people who are working in secret against the rest of us."

o Strongly disagree

o Somewhat disagree

o Neither agree nor disagree

o Somewhat agree

o Strongly agree

**News Headline Ratings**

On the following pages, you’ll be shown a series of 12 news headlines like you might see online about events from 2018. It’s possible you will have seen some or all of these before, and possible you won't have seen any – it is fine either way.

For each news story, you'll be asked to rate how it affected you.

There will be space at the end of the survey for comments, where you can explain any of your answers if you wish or state if anything was unclear.

Please read the following headline and then answer the questions about it.

[Insert headline; see end for all used]

How interesting is the story in the above headline?

o Not at all interesting

o Slightly interesting

o Moderately interesting

o Very interesting

o Extremely interesting

How much truth do you think there is to this story?

o Completely false

o Mostly false

o About half true

o Mostly true

o Completely true

[Repeated for all headlines]

Thank you! That is all the questions we have for you today. Please keep in mind that we presented headlines that were both true and false - all headlines that were not true were noted as such.

Remember that you will be getting a follow-up survey through Mturk in about two weeks that you agreed to participate in. You will be paid for your time in both surveys.

If you have any general comments to share or answers you want to explain, you may write them here if you wish.

________________________________________________________________

**Headlines used in Part 1**

True Democrat-Friendly news

**
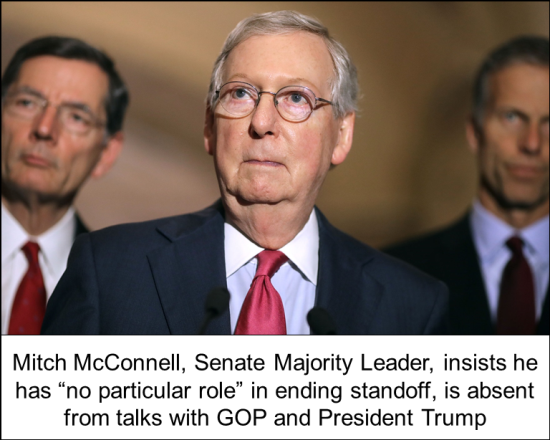

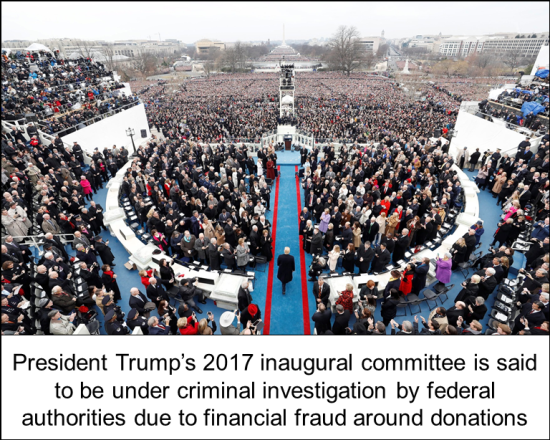
**

**
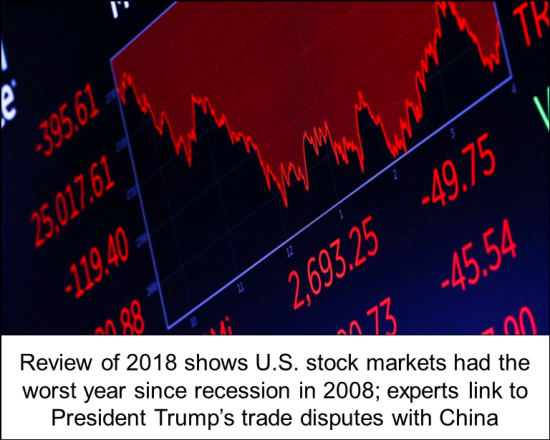

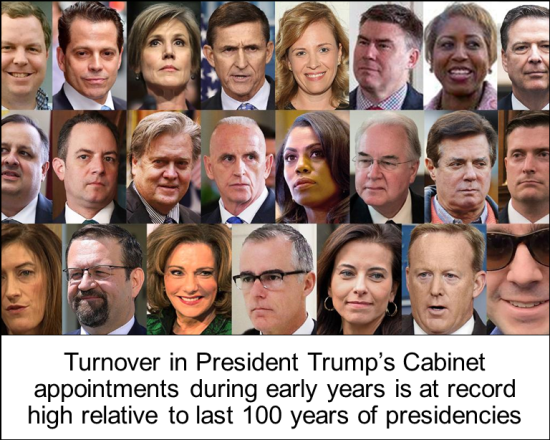
**

True Republican-Friendly News

**
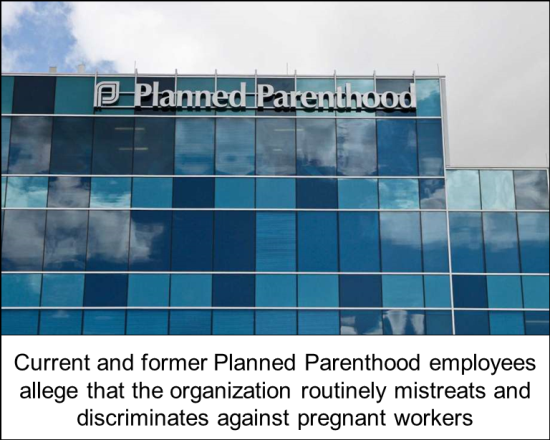

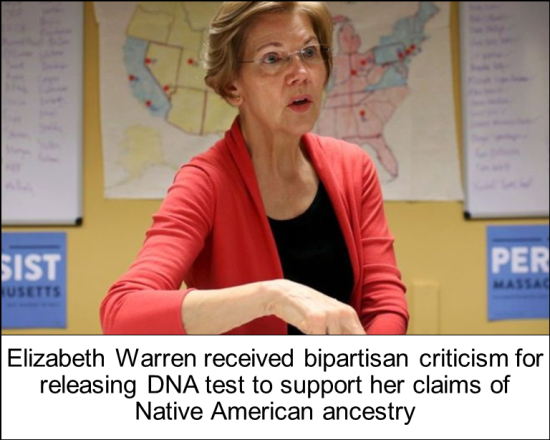
**

**
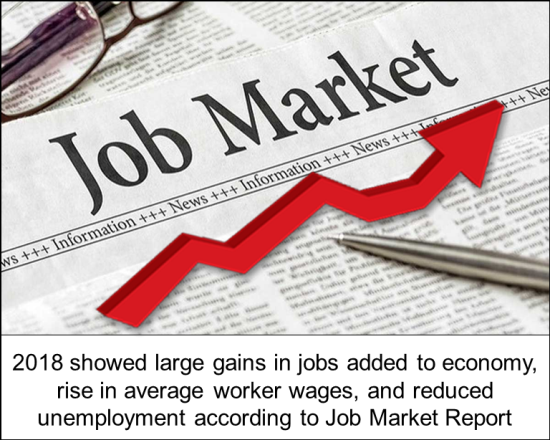

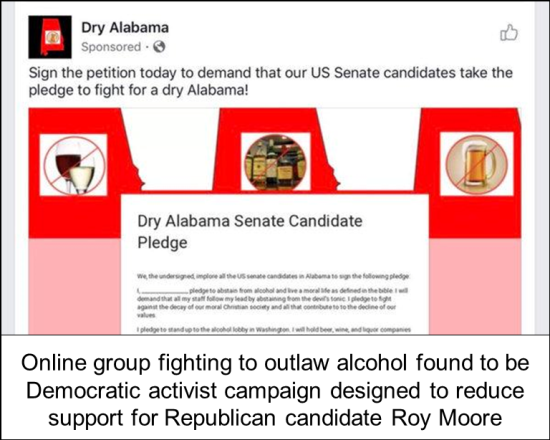
**

True Non-Partisan News

**
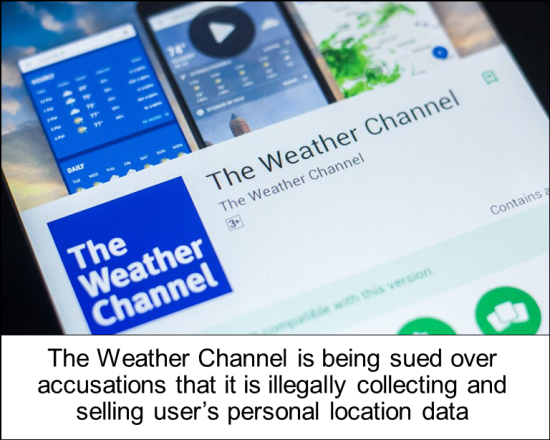

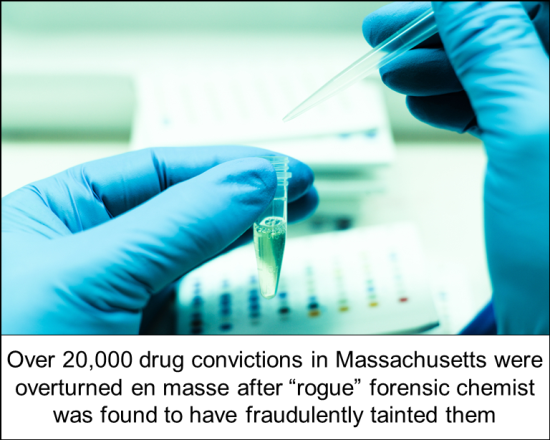
**

**
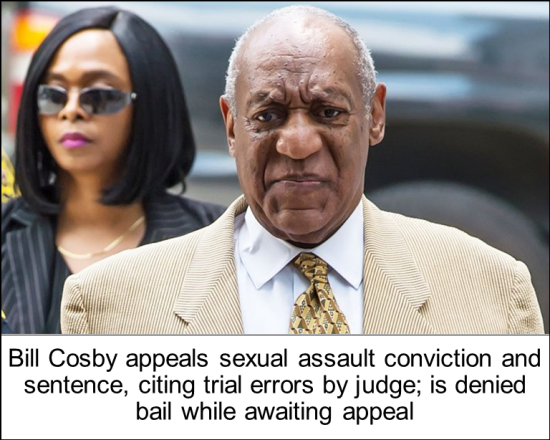

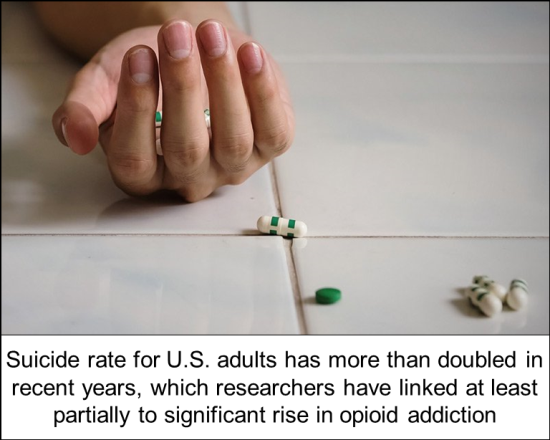
**

False News Headlines

Democrat-Friendly Fake News

**
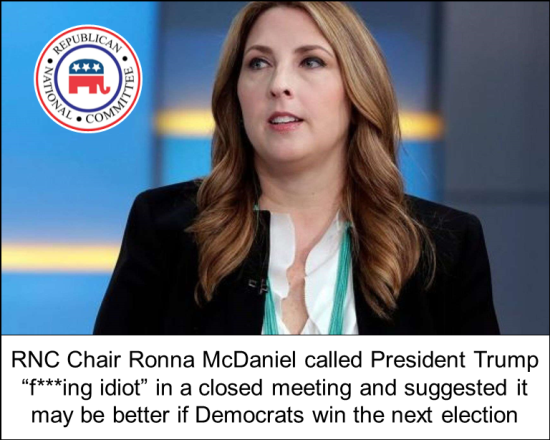
**

Republican-Friendly Fake News

**
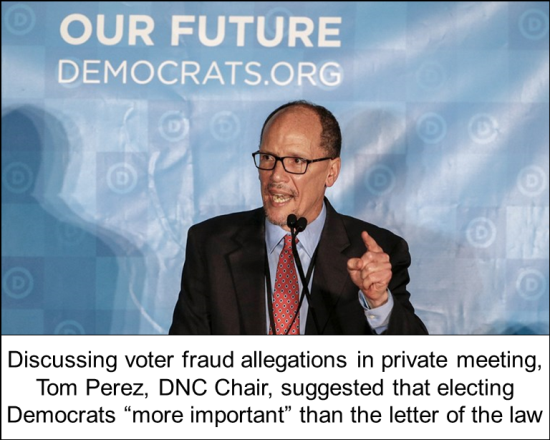
**

Non-Partisan Fake News

**
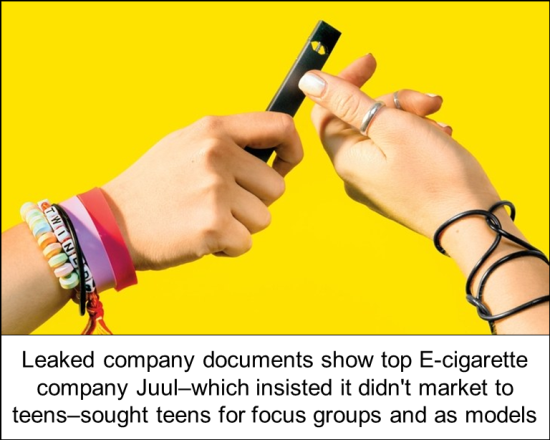
**

**Time 2 Follow-Up Materials**

Thank you for coming back to take the follow-up survey! It is similar in length and content to the first survey. We expect it to take about 10 minutes.

How much do you feel that you have followed the news in the past two weeks since last survey?

o Not at all

o A little

o A moderate amount

o A lot

o A great deal

How much do you feel that you have used social media in the past two weeks since last survey?

o Not at all

o A little

o A moderate amount

o A lot

o A great deal

On the next pages, you will be asked to again rate 16 headlines as if they were news stories you came across online. Some of them are the same as from the last survey, while others are new. You may have seen some of these in the news before now; it is fine whether or not you have. Please just give your best judgment of each one without looking up other information online.

Please read the following headline and then answer the questions about it.

[Insert headline]

How interesting is the story in the above headline?

o Not at all interesting

o Slightly interesting

o Moderately interesting

o Very interesting

o Extremely interesting

How much truth do you think there is to this story?

o Completely false

o Mostly false

o About half true

o Mostly true

o Completely true

Do you remember seeing this headline in the Part 1 survey of this study two weeks ago?

o No

o Unsure

o Yes

Have you seen or heard anything about this story outside of this survey? (e.g. heard about it from a friend, saw it on the news, read about it on social media, etc.)

o No

o Unsure

o Yes

[Repeat for all headlines]

[If relevant] You reported seeing this story outside of the survey: "RNC Chair Ronna McDaniel called President Trump “f***ing idiot” in a closed meeting and suggested it may be better if Democrats win the next election."

Can you briefly share more about where you heard it and what you felt about it?

________________________________________________________________

[If relevant] You reported seeing this story outside of the survey: "Discussing voter fraud allegations in private meeting, Tom Perez, DNC Chair, suggested that electing Democrats “more important” than the letter of the law."

Can you briefly share more about where you heard it and what you felt about it?

________________________________________________________________

[If relevant] You reported seeing this headline outside of the survey: "Leaked company documents show top E-cigarette company Juul–which insisted it didn't market to teens–sought teens for focus groups and as models."

Can you briefly share more about where you heard it and what you felt about it?

________________________________________________________________

**Self-Awareness Questions**

In the first study, you received a warning attached to some articles saying that were false. How effective do you think a warning like the one you saw would be in getting you to distrust a headline on social media?

o Not effective at all

o Slightly effective

o Moderately effective

o Very effective

o Extremely effective

o N/A - I don't remember the warning I received

In general, how accurately do you think you are able to recall whether a particular piece of news you heard is true or false?

o Not accurately at all

o Slightly accurately

o Moderately accurately

o Very accurately

o Extremely accurately

When you see a headline online, how often do you seek additional information to confirm whether it is true or false?

o Never

o Rarely

o Sometimes

o Often

o Almost always

If you come across a news story, are you more likely to seek out additional information about it if it supports your political views (e.g. something positive about your party or negative about an opposing politician) or if it goes against your political views (e.g. something negative about a politician of your party or positive about an opposing party)?

o More likely to seek additional information about a news story that supports my views

o Equally likely to seek information about a story whether or not it supports my political views

o More likely to seek additional information about a news story that goes against my views

**True or False Judgments**

Some of the headlines presented in these two surveys were false and had associated warnings explaining as such, while those without a warning were created as a composite from multiple real headlines from mainstream sources.

Out of the headlines you saw over these past two surveys, we would like you to decide whether you think it's more like that it is mostly a true store or if it is mostly a false story. Please do not do any outside searches, just use your own intuition and your memory of these stories from this survey or other things you've seen. We recognize some stories can have a mix of truth and false; please judge whether on the whole you think the claim in general is true or false.

Do you believe the claim in this headline is overall true (or mostly true) or overall false (or mostly false)?

True False

[Insert all headlines used in studies, without pictures]

**Debrief**

This study was seeking to compare the effectiveness and perceptions of different types of warning labels for false news stories. The three headlines you received a warning about in Part 1 were all entirely made up by the researchers for this study. Whether or not you remembered which were the false stories, this will help us better understand what types of warnings are more effective.

As a reminder, the below headlines all had a warning labeling them as false in part 1, and were entirely made up by the researchers for the purpose of this study:

RNC Chair Ronna McDaniel called President Trump “f***ing idiot” in a closed meeting and suggested it may be better if Democrats win the next election

Discussing voter fraud allegations in private meeting, Tom Perez, DNC Chair, suggested that electing Democrats “more important” than the letter of the law

Leaked company documents show top E-cigarette company Juul–which insisted it didn't market to teens–sought teens for focus groups and as models

Additionally, this headline in Part 2 was taken from a satire site and is not true:

A couple in California named their newborn child “😍😍😍”, the first U.S. child to have emojis on their official birth certificate

All the rest of the headlines were from credible mainstream news sources, though we cannot completely verify their veracity.

Please click below to acknowledge that you read the above information and recognize those four stories were false.

o Yes, I read the above information and recognize those headlines are not true.

Thank you! That is the end of this study. If you have any comments to share about your feelings on the warnings you received, or factors that make you seek out additional information about news, or anything else you'd want to share about fake news online, please let us know here:

________________________________________________________________

**News Headlines Added to Part 2**

True Democrat-Friendly

**
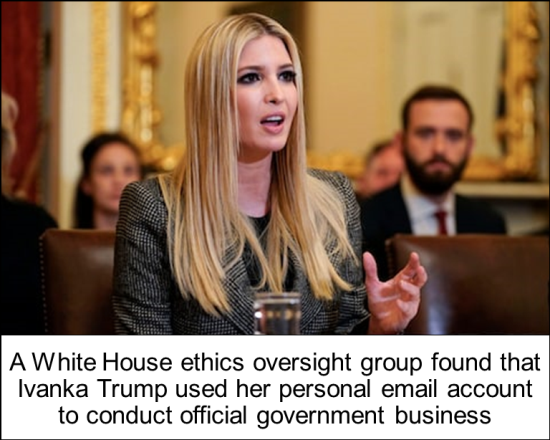
**

True Republican-Friendly

**
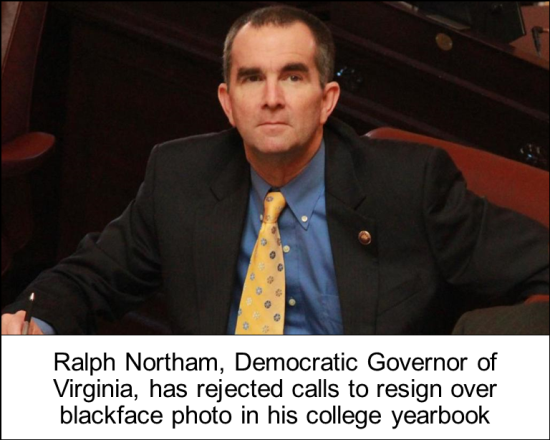
**

True Non-Partisan

**
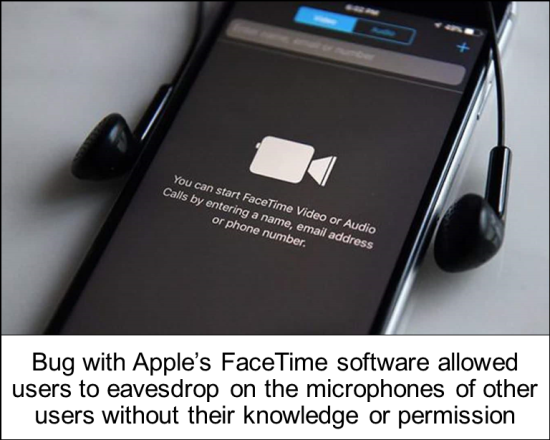
**

False Non-Partisan

**
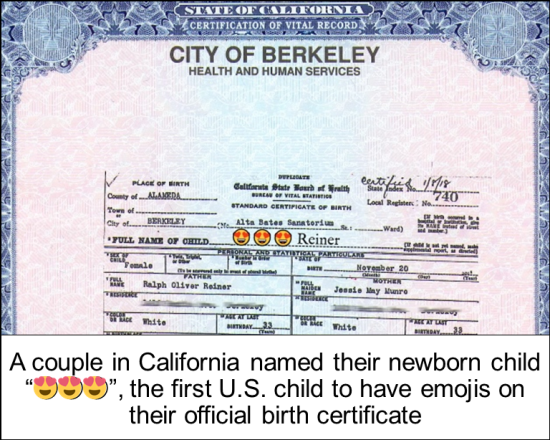
**

# Appendix C – Pilot Study to select items

To help decide on options for the political false news items for the study, a survey was posted to reddit.com/r/SampleSize, a forum where people post survey requests for anonymous respondents. To maintain privacy, no demographics were collected other than political party, where they could identify or lean towards either Democrat or Republican, or state that they did not lean towards either. There were 100 respondents in all: 71 Democrats, 13 Republicans, and 16 non-leaning independents^[[1]](#footnote-1)^. Figure C.1 below shows the items and how they were rated by each group.

Participants were given a list of 16 headlines, some that Democrats would find appealing and some that Republicans would find appealing^[[2]](#footnote-2)^, and they were told upfront that all were completely false. For each, they were asked to rate on a 5-point scale how believable each story would be if they didn’t know it was false. The top one of each was chosen for the study based on high overall believability and small differences between parties; even though all of the headlines were more believable to the side they were friendly towards, these two were less far apart than others. Other discarded headlines, for example, may have been highly believable to one party but not to another. This would have added to the difficulty in disentangling politically-motivated biases from just expectation bias. The two political stories were also relatively well-matched in content, involving a person in charge of the party’s national organization making a private comment that would be quite negative for them if made public, and also fit with general accusations from the other side that have been made in the real world. Unpublished data from a separate study (by researchers Debra Lindsay, Jacob Rode, and Peter Ditto) also found the chosen items had similar levels of plausibility and were seen as favorable to the intended group.


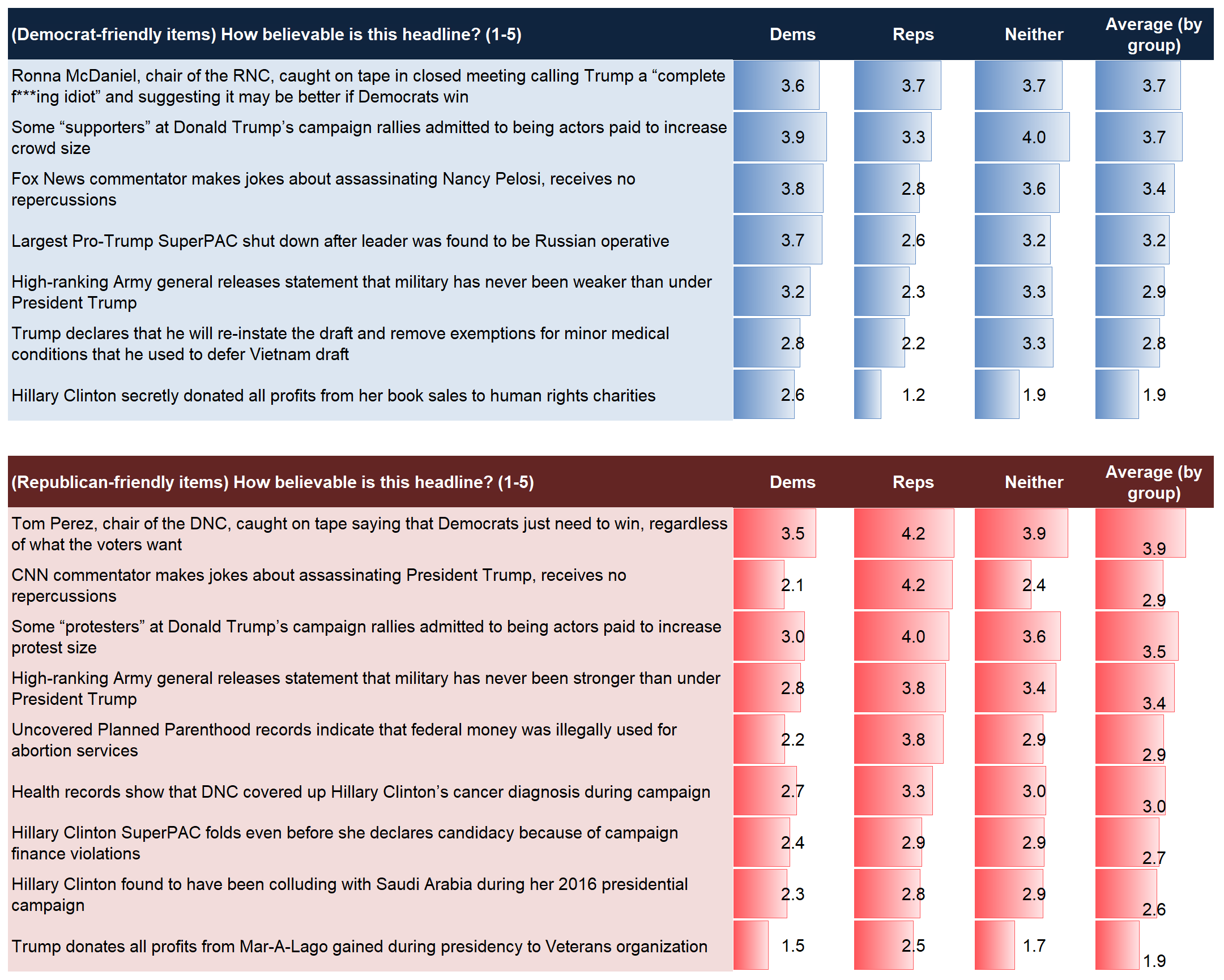


*Figure C.1*. Results by political leaning from headline selection pilot. The top item of each was chosen to use because they showed good overall believability (especially by the opposing party), had small difference between political parties, and were thematically well-matched with each other.

# Appendix D – Correlation Table for individual variables

|  | Count False Items Believed | Ideology (higher is more conservative) | Interest in Politics | Count True Items Believed | Trust in Social Media | Trust in Online News | Social Media Usage | Conspiratorial Thinking |
| --- | --- | --- | --- | --- | --- | --- | --- | --- |
| Count False Items Believed | -- |  |  |  |  |  |  |  |
| Ideology (higher is more conservative) | -0.01 | -- |  |  |  |  |  |  |
| Interest in Politics | -0.01 | -0.1 | -- |  |  |  |  |  |
| Count True Items Believed | 0.22*** | -0.13 | 0.20*** | -- |  |  |  |  |
| Trust in Social Media | -0.05 | -0.05 | 0.04 | -0.19** | -- |  |  |  |
| Trust in Online News | -0.08 | -0.11 | 0.15 | -0.10 | 0.40*** | -- |  |  |
| Social Media Usage | 0.03 | -0.12 | 0.16 | -0.01 | 0.31*** | 0.11 | -- |  |
| Conspiratorial Thinking | 0.19** | 0.15 | 0.00 | -0.03 | 0.16 | -0.07 | 0.10 | -- |

* significant at *p* = .05

** significant at *p* = .01

*** significant at *p* = .001

# Appendix E – Other analysis not included in main paper

We tested some individual moderators of the warning effectiveness and change over time that were not included in the main manuscript. These were done using a similar linear mixed regression, with random effect for intercept for each participant, and a three-way interaction between warning condition, time, and the moderator variable. Warning-Before was used as the reference group for the warning condition to compare it to both Warning-After and Warning-During, while time was a binary variable of 1 or 2. This found that gender, conservatism, and interest in politics had no significant main interaction (all *p*s > .05), meaning they did not moderate the effectiveness of the warning over time.

Conspiratorial thinking showed a significant three-way interaction for the Warning-After group (*b* = 0.188, *SE* = 0.084, *p* = .025). Following up with a separate two-way interaction between warning condition and conspiratorial thinking at each timepoint found that there was no impact of conspiracy on warning effectiveness at time 1, but that at time 2, the higher people were on the conspiracy item, the lower their fake news belief was in the warning-before condition relative to the warning after condition (but not compared to warning during). Looking at Figure E.1 below shows that the Warning-Before may have been somewhat more effective over time for those highest on the conspiracy scale, as they did not show as much of an elevation as other conditions relative to their lower conspiracy counterparts.

*Figure E.1.* Rating of fake news headline accuracy by condition, time, and level of belief in global conspiracies on the single-item measure.

We also found an effect of trust in social media, such the more people reported trusting information found on social media, the larger the difference in accuracy in judgment between the Warning-Before condition and both the Warning-During condition (*b* = 0.373, *SE* = 0.167, *p* = .020) and Warning-After condition (*b* = 0.386, *SE* = 0.180, *p* = .038). The belief in the fake news accuracy from each condition by trust in social media are presented in Figure E.2 below, and show that those with the most trust in social media information in general may be most positively affected by the strong forewarning.

*Figure E.2.* Rating of fake news headline accuracy by condition and self-reported trust in information found on social media.

Follow-up analyses showed that for while the difference between the Warning-Before and Warning-After condition was significant across all levels of trust in social media (all *p*s < .001), for those that were higher in trust in information on social media, the Warning-Before condition was also more effective than the Warning-During condition (*b* = 0.749, *SE* = 0.165, *p* < .001), a pattern not seeing in those that reported lower belief in social media (*b* = 0.111, *SE* = 0.067, *p* = .098). This could be that the warning was most effective in getting people who general trust information to be more skeptical than they otherwise would have, or their high belief may mean that they trusted the warning more, and it being especially salient was helpful in getting through to them.

These subgroup analyses are exploratory and imperfect, as they relied on single item measures and offered only a quick look at how they affected warning acceptance. They are not meant as solid conclusions but offer interesting avenues for follow-up research more focused on individual differences. In particular, if they serve to identify groups that are more amenable to warnings, this could help identify the best types of interventions that are effective with broader groups or the more relevant groups (i.e., finding the people that share the most and ensuring warning tags are effective for that group).

When looking at participant’s rating of Interestingness of the articles as a moderator of the interaction between warning condition and time, we found a significant interaction between interest and time (*b* = 0.163, *SE* = 0.060, *p* = .006), such that belief in the accuracy of false articles increased more between Time 1 and Time 2 for articles rated as more interesting.

**Belief in Fake News Truth at End of Survey**

We also analyzed the item judgments made at the end of the survey, where participants saw a list of all the news items they had seen and had to make a binary judgment on each one of them as to whether the stories were more likely true or false. Table E.1 below shows the percent believing each item by group and condition.

Table E.1

*Final belief in fake news by experimental condition and political party*

|  | Percent believing **Politically Neutral** false news as true | Percent believing **Democrat-friendly** false news as true | Percent believing **Republican-friendly** false news as true |
| --- | --- | --- | --- |
| *Experimental Condition* |  |  |  |
| Warning-Before | 63% | 32% _a_ | 27% |
|  |  | *** |  |
| Warning-During | 62% | 51% _a_ | 29% |
|  |  |  |  |
| Warning-After | 65% | 43% | 36% |
|  |  |  |  |
| *Political Party* |  |  |  |
| Democrat | 66% | 47% _b_ | 23% _cd_ |
|  |  |  |  |
| Non-leaning Independent | 57% | 38%  *** | 44% _c_  *** |
|  |  |  |  |
| Republican | 60% | 33% _b_ | 44% _d_ |
|  |  |  |  |

*Note*: Overall categories in brackets showed significant group differences (*** indicates *p*<.001 in χ^2^ tests), meaning either the experimental condition (top 3 rows) or political party (bottom 3 rows) had a significant overall impact on the rate of believing the false items. Within those groups, items with the same subscript letter were significantly different from one another at *p* < .05

We looked at each item individually in a series of chi-square analyses in order to check whether all items showed similar rates across conditions, or if it were possible that some items showed different impacts. We did find some differences in individual items. In particular, the Democrat-friendly false news item did show an effect of warning condition (χ^2^(2) = 9.660, *p* = .008), driven by those in the Warning-Before condition having a lower rate of belief in the news than those in the Warning-During condition (χ^2^(1) = 8.807, *p* = .003; the other two post-hoc comparisons did not show a significant difference). This pattern did not appear for the other two items. Additionally, there was the expected congruency effect for belief in both political news items, (*p*s < .05) in showing a difference in rate of belief based on political party that was not present for the politically neutral item (*p* = .340). There were no significant interactions between political party and warning condition for any item, meaning that partisanship was a predictor of whether people believed the item or not, and none of the warnings significantly reduced its impact.

**Order Effects: Exposure Impact and Self-Reflection**

In the Time 1 survey there was a section asking participants for their feelings towards political groups, their likelihood to vote, and the trust they have in various news sources to test whether any of these measures differed based on whether this section came at the beginning or the end of the survey. We wanted to assess whether exposure to the news items (both true and false, and mostly negative) would impact people’s responses, for example by decreasing trust in online news or increasing intention to vote. There were no significant differences by order condition on any variable (all *p*s > .05, assessed by multiple ANOVAs).

In the Time 2 survey, after rating the headlines, we varied whether participants first responded to a series of self-awareness questions about their own biases and memory (including how accurate they thought their memory was, how often they seek more information about online news stories, and how effective they thought their warning was), or first guessed which of the 19 headlines were true and which were false (with the other section coming directly after). There was no significant impact on the number of false items rated as true (*F*(1, 414) = 1.262, *p* = .262) or the number of true items rated as true (*F*(1, 413) < .001, *p* = .997) depending on whether participants had first judged their own memory and bias or not. This means that thinking first about bias, memory, and fake news warnings did not make people more skeptical or discriminating in the judgment of news accuracy.

1. While this is relatively unbalanced, it was sufficient to see clear differences in the headlines between groups and select the ones that were the most *broadly* believable, meaning they were generally believable both to people who the news was friendly towards and those to whom it was not. [↑](#footnote-ref-1)
2. Due to an error in initial categorization of items, there were 9 Pro-Republican headlines and 7 Pro-Democrat headlines tested, rather than 8 and 8. The items were intended to be loosely matched across parties (e.g., one about paid protestors against Trump, and one about paid rally attendees of Trump), and mistakenly had two negative items about Hillary Clinton in a row instead of another against Trump. [↑](#footnote-ref-2)
